# Rapid authentication of the precious herb saffron by loop-mediated isothermal amplification (LAMP) based on internal transcribed spacer 2 (ITS2) sequence

**DOI:** 10.1038/srep25370

**Published:** 2016-05-05

**Authors:** Mingming Zhao, Yuhua Shi, Lan Wu, Licheng Guo, Wei Liu, Chao Xiong, Song Yan, Wei Sun, Shilin Chen

**Affiliations:** 1 Institute of Chinese Materia Medica, China Academy of Chinese Medical Sciences, Beijing 100700, China; 2 Medical Laboratory College, Beihua University, Jilin, 132013, China; 3 Institute of Disease Control and Prevention, Academy of Military Medical Science, Beijing 100071, China

## Abstract

Saffron is one of the most expensive species of Chinese herbs and has been subjected to various types of adulteration because of its high price and limited production. The present study introduces a loop-mediated isothermal amplification (LAMP) technique for the differentiation of saffron from its adulterants. This novel technique is sensitive, efficient and simple. Six specific LAMP primers were designed on the basis of the nucleotide sequence of the internal transcribed spacer 2 (ITS2) nuclear ribosomal DNA of *Crocus sativus*. All LAMP amplifications were performed successfully, and visual detection occurred within 60 min at isothermal conditions of 65 °C. The results indicated that the LAMP primers are accurate and highly specific for the discrimination of saffron from its adulterants. In particular, 10 fg of genomic DNA was determined to be the limit for template accuracy of LAMP in saffron. Thus, the proposed novel, simple, and sensitive LAMP assay is well suited for immediate on-site discrimination of herbal materials. Based on the study, a practical standard operating procedure (SOP) for utilizing the LAMP protocol for herbal authentication is provided.

Human cultivation and use of saffron spans more than 3,500 years and extends across cultures, continents, and civilizations^1^ with documentation appearing as early as the 7^th^-century BC in an Assyrian botanical reference compiled under Ashurbanipal^2^. Saffron is a spice derived from the dried stigmas of the plant *C. sativus* that has remained among the world’s most expensive substances throughout history^3^. The apocarotenoid-rich saffron has been used as seasoning, fragrance, dye, and medicine, owing to its bitter taste, hay-like fragrance, and slightly metallic notes. It has been traded over the course of four millennia and has been used as treatment for certain disorders, such as cramps, asthma, menstruation disorders, liver disease, and pain for more than 2,500 years^2^. Chemical analysis of saffron extracts has revealed that its characteristic compounds are ds-crocins and crocetin, including carotenoid, crocetin, crocins, and monoterpene aldehydes of picrocrocin and safranal^1^,^3^,^4^,^5^. These compounds have medicinal attributes, such as strong antioxidant effects and free radical scavenging properties, as well as potential anti-carcinogenic, anti-mutagenic, anti-depressant, and memory-enhancing features that improve mood^3^,^4^. Thus, given saffron’s limited production and high price, many types of contaminants and imitations have been used to mimic saffron over the centuries. Although these imitations have similar appearances to saffron, they are cheaper and do not have the same medicinal value, thus making it difficult for consumers to immediately distinguish them. According to previous reports, the plant materials most frequently used to adulterate saffron are *Carthamus tinctorius* petals (safflower)^6^, *Curcuma longa* powdered rhizomes (turmeric)^6^, *Calendula officinalis*^6^, *Daucus carota*^6^, *Zea mays*^6^, and *Nelumbo nucifera*^6^. Previous methods used to authenticate saffron have involved physical, chemical, and molecular fields, such as ^1^H NMR metabolite fingerprinting^7^, two-dimensional gas chromatography^8^, SCAR markers^9^,^10^,^11^, and melting curve analysis^6^. These methods can identify saffron and its adulterants more quickly and accurately than traditional morphological detection. However, these processes require professional operation and correlation analysis instruments; therefore, a simple, immediate, and sensitive method for identifying adulterated saffron is required.

The loop-mediated isothermal amplification (LAMP) method is a novel nucleic acid amplification technology that is quick, simple, and highly specific. LAMP was established by Notomi in 2000^12^ and is based on a complex methodology requiring four to six different primers that are specifically designed to recognize six to eight precise gene sequences. DNA amplification is accomplished by a DNA polymerase with strand-displacing activity^13^,^14^, thus obviating the need for a thermal denaturation step to obtain single-stranded DNA. Thus, strand-displacing activity allows for amplification under isothermal conditions, in contrast to PCR (polymerase chain reaction), in which a thermal denaturation step is essential. The use of isothermal conditions in the LAMP technique allows for reactions to occur in less time because no temperature changes are required. Compared with that of PCR, the detection limit of LAMP is much lower, and only a few copies are needed^15^. Additionally, there is no need for expensive and complicated equipment, such as a thermal cycler. Because of its sensitivity, specificity, efficiency, rapidity, and simplicity, the LAMP method is an ideal molecular technique for identifying certain infectious diseases^16^,^17^ and conducting food hygiene quality control^18^,^19^, inspection, and quarantine^20^,^21^. A number of reports have demonstrated that LAMP may be useful for the detection of some plants and medicinal herbs^22^,^23^,^24^,^25^; however, a standard operating procedure (SOP) for on-site molecular authentication of Chinese herbs has not yet been established. DNA barcoding is currently an effective and widely used tool that enables the accurate identification of plant species. The ITS2 region has proven to be a core DNA barcode for authentication of a broader range of medicinal plant taxa with high variability and species-discrimination^26^. These characteristics make ITS2 a good bar-code gene choice for LAMP-specific primer design^27^. This study sought to establish a simple, rapid, and sensitive method to distinguish saffron from its adulterants by using LAMP based on the ITS2 sequence and to provide a practical SOP model for herbal medicine detection.

## Results

### Multiple alignments of *C. sativus* with other species’ ITS2 sequences

**Figure 1:**
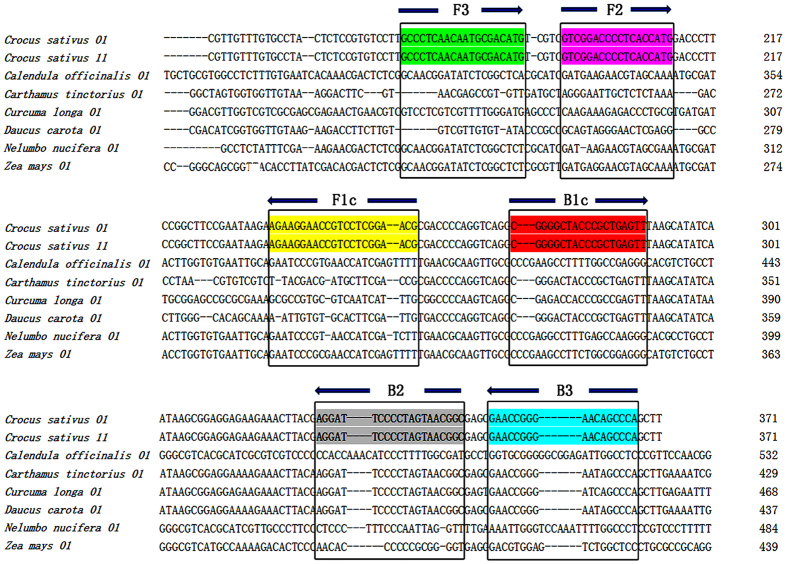
Sequence alignment of the ITS2 of *C. sativus* and its adulterants. The six ITS2 sequences of the adulterants are aligned against *C. sativus*. The sequences in coloured boxes indicate the regions that share the specifically designed LAMP primer sequences. The arrow symbols indicate the direction of DNA polymerization from the LAMP primers.

Our results showed that no variation at the site of the ITS2 sequence between the original plant *C. sativus* and the dry herb saffron. The interspecies variation of ITS2 among saffron and its adulterants ranged from 22.7% to 45.6%. On the basis of the interspecies and intraspecies sequence variation of ITS2, six specific LAMP primers were designed, F3, B3, FIP, BIP, LF, and LR (Table 1). The sequences and directions of F3, B3, F1c, and B1c are shown in Fig. 1. The sequence of FIP was combined from F3 to F1c, and BIP was linked from B3 to B1c. LF and LR were constructed according to F3, B3, F1c, and B1c.

### Authentication by LAMP amplification using specific primers

**Figure 2:**
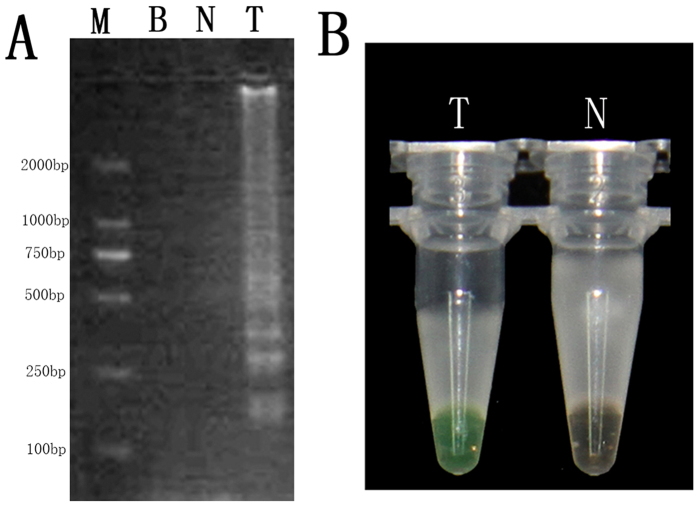
Detection of the LAMP products of *C. sativus* by using DNA electrophoresis (**A**) and calcein dyeing (**B**). The LAMP reaction was performed with the specifically designed primers. Lane B, lane N, and lane T in (**A**) represent the blank lanes, the CK line (LAMP product without template DNA), and the positive amplification lane (LAMP product with template DNA), respectively. Lane M had 2 kb of DNA ladder marker; Tube N (without template DNA) and Tube T (with template DNA) in (**B**) represent the LAMP products stained with calcein dye.

The LAMP products that were detected are shown in Fig. 2. The LAMP product showed the presence of a specific smear pattern of DNA in the gel electrophoresis in lane T. However, no such smear pattern emerged in the CK lane (LAMP product without template DNA, lane N) or the blank (lane B) (Fig. 2A). The LAMP product was directly visualized in tube T, owing to the detectable colour change to green after amplification, whereas no colour change occurred for CK (tube N) (Fig. 2B). These results indicated that the saffron LAMP primers designed were suitable for rapidly amplifying the target nucleic acids of saffron under isothermal conditions.

**Figure 3:**
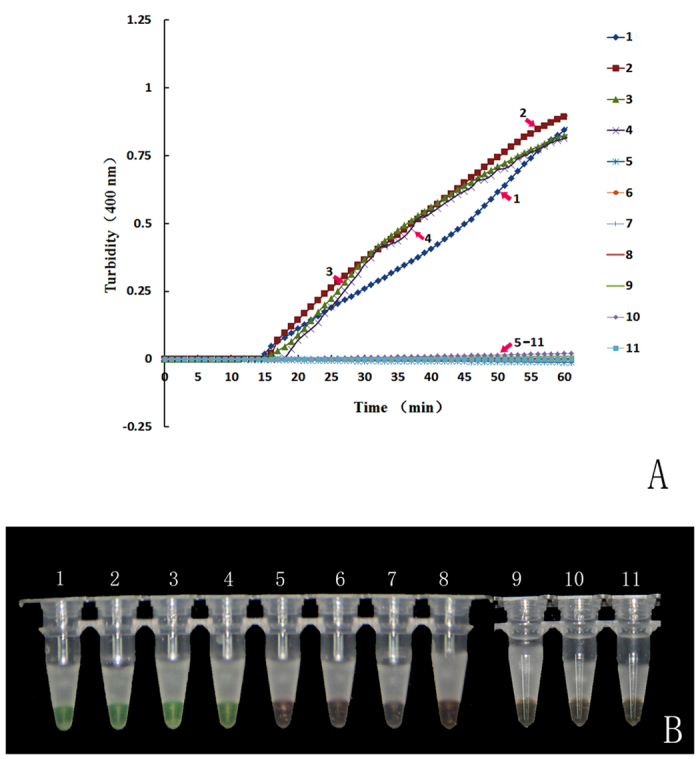
Specificity of the LAMP assay for the authentication of saffron and its adulterants. (**A**) Turbidity was monitored with a Loopamp real-time turbidimeter at 400 nm every 6 s. (**B**) A visual colour change detection method was compared. 1 μl of calcein (a fluorescent detection reagent) was added to 25 μl of the LAMP reaction mixture before the LAMP reaction. Lines and tubes: 1, *C. sativus* 01; 2, *C. sativus* 11; 3, *C. sativus* 12; 4, *C. sativus* 13; 5, *C. officinalis* 01; 6, *C. tinctorius* 01; 7, *C. longa* 01; 8, *D. carota* 01; 9, *N. nucifera* 01; 10, *Z. mays* 01; and 11, Neg (negative control, ddH_2_O).

Figure 3A indicates that the LAMP products (tubes 1–4) were initially detected within the first 15 minutes of the process and increased in concentration thereafter to 60 minutes. By the end of the reaction, the turbidity of the products of tube 1 to tube 4 reached 0.9 to 1, whereas the non-LAMP reactions (tubes 5–11) did not produce amplification curves. Figure 3B indicates that samples of original saffron plant matter and dried saffron both changed from orange to green with the addition of the templates, whereas the other tubes with non-targeted adulterant DNA remained orange after amplification for 60 minutes. Our results indicated that the LAMP method with the specific primers designed in this study can be used to rapidly discriminate saffron from its common adulterants under isothermal conditions within 60 min with the addition of calcein.

### Sensitivity of LAMP for authentication of saffron

**Figure 4:**
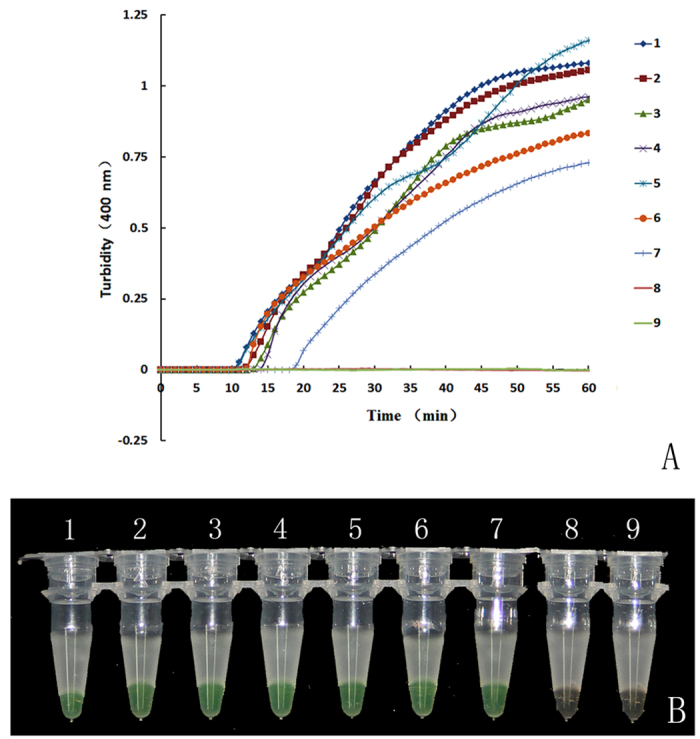
Sensitivity of the LAMP reaction for the detection of saffron. The pure genomic DNA extracted from saffron was diluted in a serial 10-fold dilution. Tubes and lanes: 1, 100 ng/μl; 2, 10 ng/μl; 3, 1 ng/μl; 4, 100 pg/μl; 5, 10 pg/μl; 6, 1 pg/μl; 7, 100 fg/μl; 8, 10 fg/μl; 9, Neg. (**A**) Turbidity was monitored with a Loopamp real-time turbidimeter at 400 nm every 6 s; (**B**) A visual colour change detection method was compared. 1 μl of calcein was added to 25 μl of LAMP reaction mixture before the LAMP reaction.

Figure 4 shows the results of the LAMP test that used saffron-specific primers with different template concentrations. The sensitivity results in Fig. 4A show that the LAMP products with template DNA concentrations of 100 ng to 1 pg (lines 1–6) began to accumulate between 9 and 14 minutes into the process. However, the template DNA concentration of 100 fg (line 7) began to amplify later, starting at 19 minutes. By the end of the test, the accumulated products of 100 fg DNA reached 0.75 turbidity. A calcein assay (Fig. 4B) supported the findings from the turbidity curves: no colour change was observed for LAMP reactions with less than 100 fg (line 8) of template DNA. The minimum amount of template used in the LAMP process to test saffron was 100 fg, thus indicating that LAMP is more sensitive than normal PCR, which typically requires a minimum template amount of 10 ng.

### A practical SOP for rapid and immediate detection of herbal medicine using LAMP

**Figure 5:**
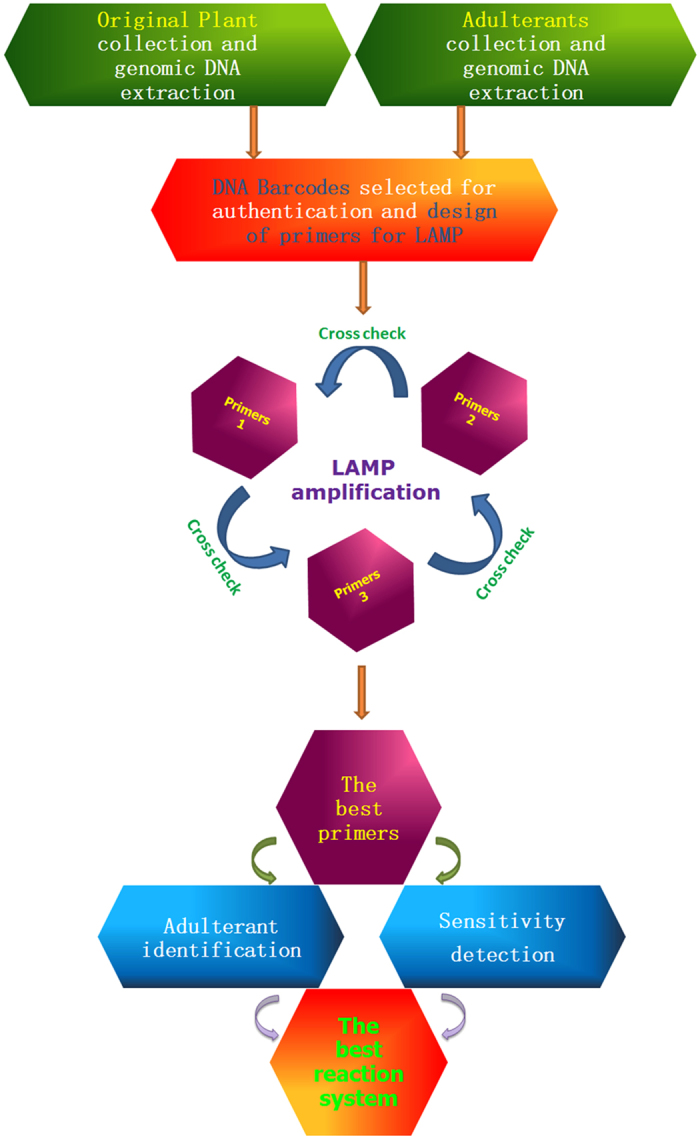
A proposed SOP for herb authentication utilizing a LAMP protocol.

The LAMP method has been used as a valid molecular technique in many fields, such as the identification of infectious diseases, testing of poisonous foods, and authentication of herbs, primarily because of its sensitivity, specificity, high efficiency, rapidity, and simplicity. The need for an immediate method of identification is urgent in the Chinese herb market. To address this, we propose a practical SOP for Chinese herb authentication utilizing the LAMP protocol (Fig. 5). First, genomic DNA from both the authentic medicinal plant and the adulterants needs to be extracted. The extracted DNA is then prepared for use as templates for PCR primers with barcodes. Second, the sections of the selected barcodes are amplified with different LAMP-specific primers designed from different barcoding sequences (i.e., ITS2, *psbA-trnH*, *rbcl*, etc.). Third, LAMP amplification is performed with different groups of primers, and then crosschecks are completed. The best group of primers that are both efficient and stable are then screened. Fourth, a preliminary test is performed to detect adulterants by using the LAMP method with specific primers for detection of the sensitivity of LAMP amplification. This fourth step focuses on the sensitivity and the identification of the authentic herb from the adulterants with each of the specific primers. Finally, the best reaction system can be constructed through the aforementioned steps.

## Discussion

This study sought to develop a rapid on-site method of discriminating the rare herb saffron (*C. sativus*) from its adulterants. Previous studies have found that saffron can be authenticated through several methods, such as barcoding-based melting curve analysis^6^, SCAR markers^9^,^10^, or ^1^H NMR metabolite fingerprinting^7^. Although these methods precisely identify species, they require professional expertise, statistical analysis, and precise instruments. Among the many methods described in previous studies, none are an efficient means for immediate authentication or can be performed by research staff without knowledge of these complex methods. From the many methods used to identify saffron from its adulterants, our results indicated that LAMP may be a superior process. Although the results of the present paper are encouraging, some problems may remain in the methodology and need to be addressed. First, because the selection of a good barcode sequence is the basis of the primer design, the fragment must be conserved, and the sequence length should be long enough to contain all of the regions adapted to designing LAMP primers. For this reason, not all common barcodes result in highly efficient LAMP primers. Therefore, we recommend designing several groups of LAMP primers based on different barcodes, such as ITS2, *psbA-trnH*, and *rbcl*. By screening the amplification efficiency of the chosen fragments, the best candidates for primers can be selected. Second, to ensure the accuracy and credibility of the test, the original plants, rather than the dry herbs, were chosen as templates to amplify the ITS2 gene, which was the target DNA for designing the specific primers for LAMP. Third, owing to the high sensitivity of the LAMP test, the occurrence of false-positive results is likely with the current experimental set-up^28^,^29^. To prevent false-positive results, we recommend using low-melting point paraffin wax to decrease the probability of contamination. In addition, spatial separation of the reagent and test samples is needed; this can be accomplished by analysing the turbidity curve in combination with the visual colour change instead of using only the DNA electrophoresis test. Finally, saffron and its adulterants can be discriminated rapidly by visual detection, but the adulterant species are not identified. To understand whether these adulterants produce toxic side effects, the traceability of the adulterants must be determined by using BLAST through the TCM barcode platform^30^, which is a part of the Traditional Chinese Medicine Database (TCMD) accessible at http://www. tcmbarcode.cn/en/31.

In summary, this study provided a novel molecular technique for rapid on-site authentication of saffron. Based on the high sensitivity of the LAMP amplification, this method is applicable for the authentication of dry herbs as well as for identification of the presence of saffron in health products. Thus, we recommend that this specific, sensitive, and convenient method be used for the commercial identification of herbal products to meet the growing need for immediate herbal authentication.

## Methods

### Plant and herbal sample collection

As shown in Table 2, we collected 31 plant samples for our analysis. 10 of these samples were pure saffron collected from Tibet, Xinjiang and Hubei province, and 21 were common adulterants collected from the Hubei and Jiangsu province and other locations. The ITS2 region is a conserved section in nuclear ribosomal DNA and is normally used as a barcode for discriminating fresh plants and dry herbs. In this work, the ITS2 sequence of these plant samples were used to design LAMP specific primers. We also purchased dried commercial herbal materials from herb markets and Taobao.com for the analysis (Table 2). Both the plant samples and the dry herb samples were used for the LAMP test. All plant samples were identified by taxonomist Wei Sun from the Chinese Material Medica, China Academy of Chinese Medical Science. All materials were stored in the herbarium of the Institute of Medicinal Plant Development of the Chinese Academy of Medical Sciences in Beijing, China.

### Genomic DNA extraction and candidate sequencing

We cut all of the plant samples and medicinal materials into small pieces of approximately 0.5 cm. We took 30 mg from each silica gel-dried plant sample and rubbed the samples for two minutes at a frequency of 30 times/second in a Mixer Mill MM400 (Retsch GmbH, Haan, Germany). The total genomic DNA was isolated using a Plant Genomic DNA kit (Tiangen Biotech Co, Ltd., Beijing, China) according to the manufacturer’s instructions. We determined the DNA concentration and purity by measuring the absorbance of a diluted DNA solution at 260 nm and 280 nm using Qubit^®^ 2.0 (Life Tech, Invitrogen, USA). To estimate the sensitivity of the LAMP assay, we prepared genomic DNA from *C. sativus* by serial 10-fold dilutions to produce concentrations ranging from 100 ng/μl to 10 fg/μl. To design the LAMP primers for the authentication of saffron, the ITS2 regions of the nuclear ribosomal DNA of *C. sativus*, *C. longa*, *D. carota*, *N. nucifera*, *Z. mays*, *C. officinalis*, and *C. tinctorius* were obtained using PCR amplification. We used the extracted genomic DNA of each sample as templates for amplifying the ITS2 sequence. The reaction mixture contained 12.5 μL 2 × Taq PCR Master Mix (Aidlab Biotechnologies Co., China), 1 μL of each primer (2.5 μmol/L), 8.5 μL ddH_2_O, and 2 μL template DNA. The conditions used for the PCR were 94 °C for 3 min, followed by 37 cycles of 94 °C for 1 min, 56 °C for 30 sec, and 72 °C for 1 min, and a final extension cycle at 72 °C for 10 min. We examined 4 μL of PCR products using 1.2% agarose gel electrophoresis. We purified the PCR products with a TIANgel Midi Purification Kit (Tiangen Biotech Co., China); then these purified PCR products were sequenced on an ABI3730XL sequencer (Applied Biosystems Inc.) using amplification primers. Sequence assembly and the generation of consensus sequences were performed with CodonCode Aligner v5.1 (CodonCode Co, USA).

### Saffron-specific primer design for LAMP

We constructed multiple alignments of the ITS2 sequence between *C. sativus* and adulterants to design saffron-LAMP-specific primers (F3, B3, FIP, BIP, LF, and LB). From the alignment results, we were able to design the LAMP-specific primers for *C. sativus* by using the online software Primer Explorer version 4.0 (http://primerexplorer.jp/elamp4.0.0/index.html). Conditions were as follows: (1) the length of F1c/B1c was 18–23 bp, whereas the lengths of F2/B2 and F3/B3 were 18–22 bp; (2) the melting temperature (Tm) for F1c/B1c was 60–65 °C, whereas the Tm for F2/B2 and F3/B3 was 58–61 °C; (3) the distance between F2 and B2 was 50–150 bp, whereas the distance between F1c and B1c was 0–50 bp. The sequences and target positions of the primers are shown in Table 1 and Fig. 1, respectively.

### LAMP amplification and product detection

The LAMP reaction was performed by using a DNA amplification kit (Eiken China Co., Ltd., Shanghai, China) in a mixture at a final volume of 25 μl, including 12.5 μl of 2× Reaction Mix (RM); 1 μl of Bst DNA polymerase; 1.6 μM of each inner primer FIP and BIP; 0.2 μM of each outer primer F3 and B3 (50 mmol); 0.8 μM of each loop primer LF and LR; 1 μl of fluorescent detection reagent (Loopamp, Eiken China Co., Ltd., Shanghai, China), and double distilled water (ddH_2_O) added to bring the reaction mix to volume. We added 1 μl of genomic DNA from each sample to each LAMP reaction as the template. The *C. sativus* 01 DNA was added as the template, and ddH_2_O was included as CK for the LAMP amplification. The mixtures were incubated at 65 °C for 60 min and 80 °C for 10 min to inactivate the Bst DNA polymerase in a loop real-time turbidimeter (LA-230; Eiken Chemical Co., Ltd., Tochigi, Japan). We monitored turbidity by recording the optical density at 400 nm every 6 s, and the addition of 1 μl of calcein (Fluorescence Detection Reagent; Loopamp, Eiken China Co., Ltd., Shanghai, China) allowed for the detection of the colour change by visual inspection. We used 2% agarose gel electrophoresis for verification of the first LAMP reaction mixture. To assess the specificity of LAMP, the saffron genomic DNA and genomic DNA from other adulterant plants were used as templates. From all of the DNA extracted, the original plant of saffron named *C. sativus* 01, the dry herbs of saffron named *C. sativus* 11, *C. sativus* 12, *C. sativus* 13, and *C. officinalis* 01, *C. tinctorius* 01, *C. longa* 01, *D. carota* 01, *N. nucifera* 01, and *Z. mays* 01 were chosen as templates for LAMP detection. To evaluate the sensitivity of the LAMP assay to detect saffron in different concentrations from its adulterants, varying amounts of extracted saffron DNA were utilized as template DNA. The amounts of extracted saffron DNA ranged from 100 ng to 10 fg.

## Additional Information

**How to cite this article**: Zhao, M. *et al.* Rapid authentication of the precious herb saffron by loop-mediated isothermal amplification (LAMP) based on internal transcribed spacer 2 (ITS2) sequence. *Sci. Rep.* **6**, 25370; doi: 10.1038/srep25370 (2016).

## Figures and Tables

**Table 1:** Primers used in this study.

Primer Name	Type	Length (bp)	Sequence (5′-3′)
ITS2	2F	ATGCTATACTTGGTGTGAAT	20
ITS2	3R	GACGCTTCTCCAGACTACAAT	21
F3	F3	GCCCTCAACAATGCGACATG	20
B3	B3	TGGGCTGTTCCCGGTTC	17
FIP	F2 + F1c	CGTTCCGAGGACGGTTCCTTCTGTCGGACCCCTCACCATG	40
BIP	B2 + B1c	CGGGGCTACCCGCTGAGTTGCCGTTACTAGGGGAATCCT	39
LF	LF	TTCGGAAGCCGGAAGGGTC	19
LR	LR	ATAAGCGGAGGAGAAGAAACTTAC	24

**Table 2:** The species origin, collection area and identifiers of saffron (*C. sativus*) and its common adulterants in this study.

Samples	Species	Plant (portion)	Crude drug (portion)	Origin	Identifier
CS01	*Crocus sativus*	Y/filament	N	Tibet China	Wei Sun
CS02	*C. sativus*	Y/filament	N	Tibet China	Wei Sun
CS03	*C. sativus*	Y/filament	N	Tibet China	Wei Sun
CS04	*C. sativus*	Y/filament	N	Tibet China	Wei Sun
CS05	*C. sativus*	Y/filament	N	Tibet China	Wei Sun
CS06	*C. sativus*	Y/filament	N	Tibet China	Wei Sun
CS07	*C. sativus*	Y/filament	N	Xinjiang China	Wei Sun
CS08	*C. sativus*	Y/filament	N	Xinjiang China	Wei Sun
CS09	*C. sativus*	Y/filament	N	Hubei China	Wei Sun
CS10	*C. sativus*	Y/filament	N	Hubei China	Wei Sun
DC01	*Daucus carota*	Y/leaf	N	Hubei China	Wei Sun
DC02	*D. carota*	Y/leaf	N	Hubei China	Wei Sun
DC03	*D. carota*	Y/leaf	N	Hubei China	Wei Sun
DC04	*D. carota*	Y/leaf	N	Hubei China	Wei Sun
DC05	*D. carota*	Y/leaf	N	Hubei China	Wei Sun
DC06	*D. carota*	Y/leaf	N	Hubei China	Wei Sun
CL01	*Curcuma longa*	Y/root	N	Guangdong China	Wei Sun
CL02	*C. longa*	Y/root	N	Jiangsu China	Wei Sun
CL03	*C. longa*	Y/root	N	Fujian China	Wei Sun
NU01	*Nelumbo nucifera*	Y/root	N	Beijing China	Wei Sun
NU02	*N. nucifera*	Y/root	N	Jilin China	Wei Sun
NU03	*N. nucifera*	Y/root	N	Jilin China	Wei Sun
ZM01	*Zea mays*	Y/leaf	N	Jilin China	Wei Sun
ZM02	*Z. mays*	Y/leaf	N	Jilin China	Wei Sun
ZM03	*Z. mays*	Y/leaf	N	Jilin China	Wei Sun
CO01	*Calendula officinalis*	Y/leaf	N	Yunnan China	Wei Sun
CO02	*C. officinalis*	Y/leaf	N	Sichuan China	Wei Sun
CO03	*C. officinalis*	Y/leaf	N	Yunnan China	Wei Sun
CT01	*Carthamus tinctorius*	Y/petal	N	Yunnan China	Wei Sun
CT02	*C. tinctorius*	Y/petal	N	Yunnan China	Wei Sun
CT03	*C. tinctorius*	Y/petal	N	Sichuan China	Wei Sun
CS11	saffron	N	Y/dry filament	TongRenTang pharmacy	
CS12	saffron	N	Y/dry filament	Anguo herb market, Hebei	
CS13	saffron	N	Y/dry filament	Taobao.com	
DC07	*D. carota*	N	Y/fresh root	Taobao.com	
DC08	*D. carota*	N	Y/fresh root	Anguo herb market	
DC09	*D. carota*	N	Y/fresh root	Bozhou herb market	
NU04	*N. nucifera*	N	Y/dry stigmas	Taobao.com	
NU05	*N. nucifera*	N	Y/dry stigmas	Anguo herb market	
NU06	*N. nucifera*	N	Y/dry stigmas	Bozhou herb market	
ZM04	*Z. mays*	N	Y/dry stigma maydis	Taobao.com	
ZM05	*Z. mays*	N	Y/dry stigma maydis	Anguo herb market	
ZM06	*Z. mays*	N	Y/dry stigma maydis	Bozhou herb market	
CO04	*C. officinalis*	N	Y/dry flowers	Taobao.com	
CO05	*C. officinalis*	N	Y/dry flowers	Taobao.com	
CO06	*C. officinalis*	N	Y/dry flowers	Taobao.com	
CT04	*C. tinctorius*	N	Y/dry stigmas	TongRenTang pharmacy	
CT05	*C. tinctorius*	N	Y/dry stigmas	YiDeTang pharmacy	
CT06	*C. tinctorius*	N	Y/dry stigmas	JiXiang pharmacy	
CL04	*C. longa*	N	Y/dry root	TongRenTang pharmacy	
CL05	*C. longa*	N	Y/dry root	YiDeTang pharmacy	
CL06	*C. longa*	N	Y/dry root	JiXiang pharmacy	

Y = Yes and N = No.
